# Associations among parental mental health, lifestyle factors and infant mortality in rural and urban mothers

**DOI:** 10.3389/fpubh.2025.1622333

**Published:** 2025-10-01

**Authors:** S. Mudasser Shah, Zijin Zhang, Muhammad Jahangir, Fatimah Sayer Alharbi, Wenrui Zhang, Xiuyun Lin

**Affiliations:** ^1^Institute of Developmental Psychology, Beijing Normal University, Beijing, China; ^2^Affiliated Mental Health Center and Hangzhou Seventh Peoples Hospital, Zhejiang University School of Medicine, Zhejiang, China; ^3^Department of Health Sciences, College of Health and Rehabilitation Sciences, Princess Nourah Bint Abdulrahman University, Riyadh, Saudi Arabia; ^4^Beijing Key Laboratory of Applied Experimental Psychology, Faculty of Psychology, Beijing Normal University, Beijing, China

**Keywords:** mental health, depression, anxiety, physical activities, diet, infant mortality, rural mothers, urban mothers

## Abstract

**Background:**

Infant mortality remains a critical public health concern, necessitating a comprehensive understanding of its determinants. This investigation aimed to examine associations between social determinants, lifestyle factors, and maternal mental health in relation to infant mortality.

**Methods:**

A cross-sectional survey was conducted among 500 mothers (250 rural, 250 urban) in Khyber Pakhtunkhwa (KPK), Pakistan. The Depression Anxiety Stress Scales (DASS-21) were employed to evaluate symptoms of anxiety, depression, and stress, while a Lifestyle and Habits Questionnaire collected data on physical activity and dietary patterns. Data was collected via questionnaires and demographic information from 500 mothers using purposive sampling. Key variables included rural/urban residence, age at marriage, socio-economic status, access to healthcare, type of delivery attendant, and under-5 mortality rates.

**Results:**

Rural mothers had significantly lower infant mortality rates (*p* = 0.000) compared to urban mothers. Physical activity and diet were negatively correlated with depression, anxiety, and stress (*p* < 0.05). Rural mothers reported higher physical activity (*M* = 23.46 vs. 21.79, *p* = 0.001) and healthier diets (*M* = 16.01 vs. 14.85, *p* = 0.001). Urban mothers exhibited significantly higher levels of depression (*M* = 6.59 vs. 1.63, *p* = 0.000), anxiety (*M* = 7.68 vs. 2.18, *p* = 0.000), and stress (*M* = 9.65 vs. 2.32, *p* = 0.000). Early marriage was linked to increased anxiety and stress (*p* = 0.000).

**Conclusion:**

Findings underscored the importance of addressing social determinants and fostering healthy lifestyles to improve maternal and child health outcomes. Interventions that promote access to healthcare, physical activity or healthy dietary habits can help make the infant mortality rates and the overall state of health of the mother better.

## Background

Infant death refers to the death of children under the age of one year and remains a key socioeconomic development indicator. UNICEF (1) reported 4.9 million child deaths, with most being infants. These are largely preventable and linked to nutrition, maternal education, essential services, and health inequalities. Globally, 90% of infant deaths occur in low- and middle-income countries where poverty and poor health infrastructure dominate (2). In South Asia, Pakistan ranks among the highest with 57 deaths per 1,000 live births, despite policy interventions (1), highlighting the need to study social factors influencing mortality.

Determinants where people are born and live like socioeconomic, environmental, cultural, income, education, and healthcare access are critical (3, 4). Poverty and low maternal education consistently predict higher mortality (5–7). Infants of uneducated mothers face greater death risk than those with educated mothers (8). Inequalities are not limited to developing nations; in the U.S., African-American newborns have higher mortality due to racialized healthcare, income gaps, and neighborhood deprivation (9–11).

Urban–rural divides remain persistent. Rural areas lack trained attendants and facilities, forcing reliance on traditional birth attendants (12, 13). Urban settings, despite better access, face overcrowding, pollution, and poor sanitation (14). In Pakistan, mountainous terrain, marginalization, and tribal norms cause delays in care, poor rural staffing, and fatal complications (15–17). Urban slums mirror many rural disadvantages (14, 18).

Maternal lifestyle is also crucial. Prenatal physical activity reduces preterm births, low birth weight, and gestational diabetes, with meta-analyses showing a 15–30% reduction in adverse outcomes (19, 20). Yet cultural barriers, safety concerns, and misconceptions restrict activity in Pakistan (21–24). Antenatal education promoting safe activity could improve outcomes. Nutrition is equally vital. Undernutrition contributes to intrauterine growth restriction, anemia, and LBW (25, 26). Over 40% of Pakistani pregnant women are anemic, and one in five underweight (27, 28). Food insecurity reduces antenatal care and nutrition counseling (29, 30). Diet diversity and supplementation with iron and folic acid remain essential (31).

Maternal mental health is a significant but often neglected determinant. Stress, anxiety, and depression predict preterm birth, poor postnatal care, and LBW (32, 33). Untreated illnesses reduce visits, breastfeeding, and bonding (34). In Pakistan, stigma and lack of services lead to underdiagnosed perinatal depression, particularly in rural areas (35). Such mothers often lack emotional support, nutrition, and antenatal care, with long-term cognitive and emotional risks for infants (36).

In Khyber Pakhtunkhwa (KPK), early marriages and adolescent childbirth pose risks including obstructed labor, hemorrhage, LBW, and infant mortality (37, 38). Girls married under 18 face reduced education, limited autonomy, and poor reproductive health access (39, 40). Nearly one-third in rural Pakistan marry before 18, worsening malnutrition and neonatal complications (41, 42). Addressing this requires legal enforcement, education, and culturally adapted outreach.

Existing studies document national determinants: disparities in Punjab (43), cultural impacts in Sindh (44), and links between maternal education, income, and outcomes (45). However, literature on KPK is scarce, despite its unique cultural practices and geographical barriers. This study therefore investigates how social determinants like maternal mental health, physical activity, and dietary patterns shape infant survival in rural and urban KPK.

### Conceptual framework: web of causation

## Methods

### Research design

**Figure 1:**
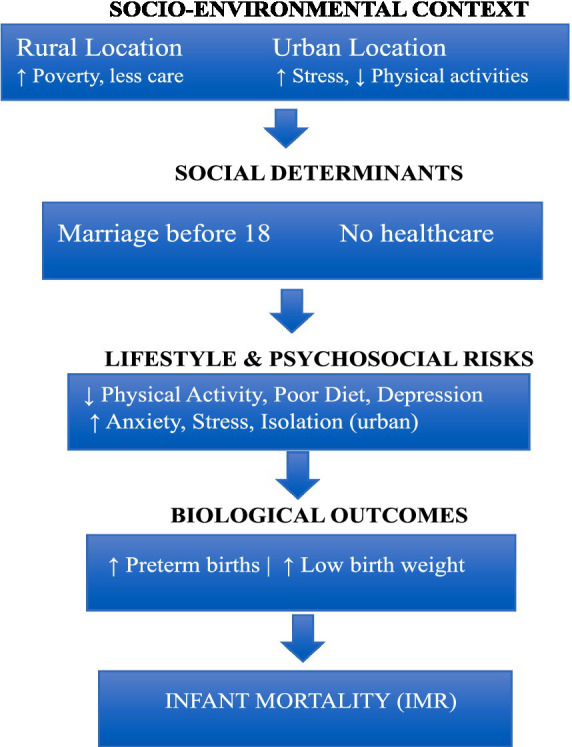
Web of causation model for infant mortality in rural and urban Pakistan (43).

The current study was correlational and cross-sectional in nature utilizing questionnaire survey targeting mothers residing in rural and urban areas of Khyber Pakhtunkhwa (KPK), Pakistan. KPK was chosen as the study location due to its distinct socio-economic, geographical, and cultural characteristics, as well as the region’s historically elevated rates of infant mortality. The study period spanned from February 15, 2024, to September 1, 2024. This design was chosen to explore potential associations between maternal lifestyle factors, mental health, and infant mortality outcomes across different settings (Figure 1).

### Population

In the study ethical guidelines were strictly adhered to, with approval obtained from the Institutional Ethics Review Board. Informed consent was collected from all participants, and confidentiality was maintained by anonymizing responses and securely storing all data to ensure privacy throughout the study. The population was mothers of rural and urban areas who had experienced infant mortality. There were 500 samples chosen via purposive sampling techniques from rural and urban areas with 250 from rural and 250 from urban areas. The inclusion criteria were mothers from rural and urban areas, mothers married before or after the age of 18 years, and participants who voluntarily agreed to participate and provide informed consent. The exclusion criteria were, excludes mothers who have had stillbirths or no live births, excludes mothers with severe uncontrolled medical conditions or psychiatric disorders. Excludes mothers who do not comply with study protocols or refuse participation and exclude participants with missing data on key variables (e.g., neonatal deaths, SES, delivery, healthcare access).

## Instruments

### Demographic sheet

Demographic information contains age, area, marriage before 18 years, Socioeconomic status (lower, middle & Higher), access to health care, attendant, delivery at home or hospital, number of neonatal deaths, and under 5 mortalities. Socioeconomic status was assessed using income categories outlined in a Dawn newspaper report dated April 2, 2024: 4,000–20,000 PKR as lower, 50,000–100,000 PKR as middle, and above as upper class (44).

### Depression Anxiety Stress Scale (DASS)

The Depression Anxiety Stress Scales (DASS-21) is a popular self-report tool for gauging the intensity of depressive, anxious, and stress-related symptoms experienced in the previous seven days. The Depression Anxiety Stress Scale (DASS-21) was created by Lovibond and Lovibond and has a Cronbach Alpha of 0.91 for Depression, 0.81 for Anxiety, and 0.89 for Stress (45). It is a valid and efficient way to measure emotional discomfort in these three areas.

### The lifestyle and habits questionnaire-B (LHQ-B)

It was developed by Dinzeo et al. (46), which has 42-items with 5-points response range strongly disagree to strongly agree. It has 8 subscales, but we used only physical activity and diet with Cronbach Alpha ranging from 0.65 to 0.91.

### Statistical analysis

Statistical methods, chi-square tests were used to examine whether there is a significant association or difference between variables and for comparison. *t*-tests were used to compare the means (averages) of two groups to see if they are significantly different from each other. These tests were used to analyze the relationships between socio-economic factors, physical activity, diet, and mental health.

## Results

The current study tended to explore social determinants of infant mortality and compared rural and urban mothers. The results generated and tabulated are given below.

Table 1 presents the characteristics of 500 mothers from Khyber Pakhtunkhwa, with a balanced representation from rural and urban areas. Key findings include a high percentage of young mothers married before the age of 18 (49.8%) and a clear trend toward middle-class socioeconomic status (54.4%) among the participants. Access to healthcare centers was notably low, with 54% of the participants reporting limited access. The majority of deliveries took place in hospitals (76.8%), and neonatal mortality was relatively low, with 79% of mothers reporting no neonatal deaths. These findings underscore the importance of healthcare access and early medical intervention in reducing infant mortality rates (Table 2).

**Table 1:** Basic characteristics of participants (*n* = 500).

Variable	Categories	*n*	%
Area			
	Rural	250	50
	Urban	250	50
Marriage under 18 years old			
	Yes	249	49.8
	No	251	50.2
Socioeconomic status			
	Upper class	181	36.2
	Middle class	272	54.4
	Lower class	47	9.4
Access to health care centre			
	Yes	230	46.0
	No	270	54.0
Attended delivery			
	Midwife	148	29.6
	Nurse	215	43.0
	Doctor	137	27.4
Delivery			
	At home	116	23.2
	At hospital	384	76.8
No. of neonatal deaths			
	0	395	79.0
	1	105	21.0
No. of post neonatal deaths			
	0	438	87.6
	1	62	12.4

**Table 2:** Social determinant of infant mortality (*n* = 500).

Social factors	Perception	Infant mortality		Total	*χ* ^2^	*p*
		Yes	No			
Area	Rural	98	152	250	51.99	0.000
	Urban	28	22	250		
Marriage before 18	Yes	91	158	249	33.88	0.000
	No	35	216	251		
Socioeconomic status	Lower	47	134	181	0.826	0.662
	Middle	65	207	272		
	Upper	14	33	47		
Access to health care center	Yes	47	183	230	5.13	0.030
	No	79	191	270		
Delivery attended	Midwife	45	103	148	3.17	0.205
	Nurse	51	164	215		
	Doctor	30	107	137		
Delivery	At home	35	81	116	1.98	0.180
	At hospital	91	293	384		
Neonatal deaths	0	99	296	395	0.019	0.900
	1	27	78	105		
Post neonatal deaths	0	108	330	438	0.551	0.439
	1	18	44	62		
Under 5 years died	0	94	272	366	0.169	0.387
	1	32	102	134		

The analysis of social determinants of infant mortality revealed significant associations with certain factors. Areas with rural populations showed significantly higher rates of infant mortality compared to urban areas (χ^2^ = 51.99, *p* = 0.000). Additionally, early marriage (before 18 years) was linked to a higher incidence of infant mortality (χ^2^ = 33.88, *p* = 0.000), and lack of access to health care centers also contributed to higher mortality rates (χ^2^ = 5.13, *p* = 0.030). On the other hand, no significant associations were found for other social determinants. Socioeconomic status did not appear to affect infant mortality (χ^2^ = 0.826, *p* = 0.662), and the type of birth attendant (midwife, nurse, or doctor) showed no significant difference in mortality rates (χ^2^ = 3.17, *p* = 0.205). Delivery location (home or hospital), neonatal deaths, post-neonatal deaths, and under-5-year deaths were also not significantly associated with infant mortality, as indicated by their respective *p*-values (all > 0.05).

Table 3 presents Pearson correlation coefficients exploring the relationships between lifestyle factors (physical activity and diet) and mental health outcomes (depression, anxiety, and stress) among rural and urban mothers (n = 500). A statistically significant negative correlation was found between physical activity and depression (*r* = −0.149, *p* < 0.01), anxiety (*r* = −0.146, *p* < 0.01), and stress (*r* = −0.135, *p* < 0.05), indicating that higher levels of physical activity are associated with lower levels of psychological distress. Similarly, diet was negatively correlated with depression (*r* = −0.139, *p* < 0.05), anxiety (*r* = −0.130, *p* < 0.05), and stress (*r* = −0.152, *p* < 0.01), suggesting that healthier dietary habits are linked to better mental health. Strong positive correlations were also observed among the mental health variables themselves: depression with anxiety (*r* = 0.660, *p* < 0.01), depression with stress (*r* = 0.623, *p* < 0.01), and anxiety with stress (*r* = 0.682, *p* < 0.01).

**Table 3:** Correlation among lifestyles (physical activity, diet), and mental health (depression, anxiety and stress) among rural and urban mothers (*n* = 500).

Variables		M	SD	1	2	3	4	5
Lifestyle	Physical activity	22.63	4.91	-				
	Diet	15.43	3.35	0.551**	-			
Mental health	Depression	5.44	3.84	−0.149**	−0.139*	-		
	Anxiety	6.40	3.38	−0.146**	−0.130*	0.660**	-	
	Stress	7.94	3.79	−0.135*	−0.152**	0.623**	0.682**	-

**Correlation is significant at the 0.01 level (2-tailed).

*Correlation is significant at the 0.05 level (2-tailed).

Table 4 shows rural mothers showed significantly better lifestyles compared to their urban counterparts. They engaged in more physical activity (*M* = 23.46 vs. 21.79, *p* = 0.001) and followed a healthier diet (*M* = 16.01 vs. 14.85, *p* = 0.001). These differences were statistically significant, indicating better lifestyle habits in rural areas. However, urban mothers reported significantly worse mental health outcomes. They experienced much higher levels of depression (*M* = 6.59 vs. 1.63, *p* = 0.000), anxiety (*M* = 7.68 vs. 2.18, *p* = 0.000), and stress (*M* = 9.65 vs. 2.32, *p* = 0.000) compared to rural mothers, with all differences being highly significant.

**Table 4:** Mean difference along area on lifestyles (physical activity, diet), and mental health (depression, anxiety and stress) among rural and urban mothers (*n* = 500).

Variable		Rural (250)		Urban (250)		*t*	*p*
		M	SD	M	SD		
Lifestyles	Physical activity	23.46	4.46	21.79	5.19	3.86	0.001
	Diet	16.01	3.05	14.85	3.54	3.92	0.001
Mental health	Depression	1.63	0.81	6.59	3.65	−11.76	0.000
	Anxiety	2.18	0.89	7.68	2.76	−17.10	0.000
	Stress	2.32	0.96	9.65	2.43	−25.66	0.000

M, mean; SD, standard deviation.

Table 5 shows that mothers who married before 18 showed no significant differences in lifestyles like physical activity (*M* = 22.84 vs. 22.42, *p* = 0.72) and diet (*M* = 15.67 vs. 15.2, *p* = 0.25) and depression (*M* = 6.08 vs. 4.32, *p* = 0.13) compared to those who married later. However, significant differences were found in mental health, with those who married early reporting higher levels of anxiety (*M* = 7.14 vs. 5.12, *p* = 0.00) and stress (*M* = 8.87 vs. 6.32, *p* = 0.00).

**Table 5:** Mean Difference along marriage before 18 on lifestyles (physical activity, diet), and mental health (depression, anxiety and stress) among rural and urban mothers (*n* = 500).

Variable		Yes (249)		No (251)		*t*	*p*
		*M*	*SD*	*M*	*SD*		
Lifestyles	Physical activity	22.84	4.854	22.42	4.961	0.96	0.72
	Diet	15.67	3.286	15.2	3.397	1.58	0.25
Mental health	Depression	4.32	3.577	6.08	3.847	−4.07	0.13
	Anxiety	5.12	3.554	7.14	3.047	−5.41	0.00
	Stress	6.32	4.235	8.87	3.168	−6.17	0.00

M, mean; SD, standard deviation.

## Discussion

In this study, the social determinants of infant mortality among rural and urban mothers were analyzed, focusing on factors such as physical activity, diet, and mental health. The result of the present study indicates that higher infant mortality tends to be correlated to rural residence and early marriage.

The descriptive data shown in Table 1 on neonatal and post-neonatal mortality is supported by recent studies emphasizing the importance of socioeconomic status, healthcare access, and delivery conditions. For instance, Izulla et al. (47) highlight that skilled birth attendance and facility delivery are critical in reducing neonatal mortality, aligning with the higher mortality rates observed for home births in the data. Similarly, Kibret (48) points out how socioeconomic disparities in healthcare access contribute to higher neonatal mortality. Additionally, Chauhan and Verma (49) confirm that skilled delivery significantly reduces neonatal and post-neonatal mortality in India. Ogbo et al. (50) also demonstrate that factors like maternal education and place of residence affect neonatal survival, with facility-based deliveries yielding better outcomes. Furthermore, Temporin (51) highlights how socioeconomic factors in Bolivia, including access to skilled birth attendants, contribute to better neonatal survival. These studies strengthen the findings from the descriptive data, indicating that socioeconomic inequalities and access to healthcare significantly impact neonatal and post-neonatal outcomes.

Our results revealed that Areas with rural populations showed significantly higher rates of infant mortality compared to urban areas (χ^2^ = 51.99, *p* = 0.000), supported by both international and Pakistani literature. Numerous studies support our findings presenting various reasons, i.e., limited access to maternal healthcare, poor infrastructure and lower literacy in rural areas. For example, limited access to skilled birth attendants in rural Ethiopia (52), studies in the United States (53, 54), and China (55, 56) consistently found that rural regions suffer from higher infant mortality due to socioeconomic disadvantages, inadequate healthcare access, and systemic inequities. These trends are echoed by Singh et al. (57), who highlighted growing disparities between Appalachia and more urban U.S. regions, and by Dagher and Linares (3), who stressed the impact of social determinants of health. However, evidence also indicates that targeted reforms can mitigate these disparities: Aidoo (58) reported reductions in mortality through community-based rural health interventions, while well-designed regional policies can significantly narrow the rural–urban mortality gap (59).

Additionally, adolescents who get married early have been found to have higher infant mortality rates due to teen pregnancy, less access to healthcare, and fewer opportunities for education due to being young mothers (60, 61). On the other hand, some studies have not demonstrated a connection between infant mortality ratios and delivery related risk factors or socio-economic status and suggest that other factors, such as age of mother and georeferential location might outweigh those (62–64). In the rural areas, mothers or women who will bear children have more unfavorable systems for healthcare such as, low income, education, and very less availability of health services to make is easier to the women to bear children so; these conditions lead to infant mortality because these women are not able to handle their pregnancy or the issues relating to pregnancy properly. These issues are now reflected as the studies on rural urban difference map these issues and have proved the existence of these issues (59, 65).

Among rural and urban mothers, our findings showed notable negative relationships between physical activity and diet with mental health indicators (depression, anxiety, and stress). Studies supporting this one have found that physical activity lowers anxiety and stress; one study found that regular exercise helps to lower postpartum depression (66, 67). Studies have also shown that a balanced diet helps to alleviate depressive and anxious symptoms in mothers (68, 69). Some studies, meanwhile, question these links and argue that other variables such as healthcare and socioeconomic level might be more important predictors of mental health results than physical activity or diet alone (70, 71). Regular exercise and good nutrition’s physiological and psychological advantages explain these correlations; they lower inflammation and enhance hormonal balance, both of which have been connected to improved mental health (70, 72).

Moreover, our findings underlined the different physical activity, dietary and mental health markers (i.e., depression, anxiety, and stress) between rural and urban mothers, with rural mothers indicating more physical activity and better diets, and lower levels of mental health distress. Previous supporting studies have found that rural mothers demonstrate healthier lifestyle behavior, such as nutrition and physical activity (both of which could related to increased community involvement and less artificial nature) (73, 74). While urban areas are often perceived as having a wider variety of health promoting resources such as gyms, and dietary diversity, as is revealed by contradictory research (75, 76), they may also actually provide a better place for mothers to engage in physical activity and nutrition despite having reported mental health issues. In explaining rural urban disparities on mental health, Singh et al. (76) attributed it to the different environmental stressors, urban mothers having greater socioeconomic stress and less access to green spaces, in comparison to rural mothers who experience other challenges of limited healthcare but report high amount of community support and less stress from urban conditions in living (77).

## Conclusion

The results showed notable links between demographic variables and newborn death; rural areas and early marriage before 18 indicated higher infant death rates. Mental health indicators including sadness, anxiety, and stress were negatively correlated with lifestyle choices including physical activity and diet. Rural mothers had better lifestyle decisions and far less mental health problems than urban mothers as well. Though it did not affect lifestyle decisions, early marriage was linked to poorer mental health results, especially anxiety and stress. Moreover, healthcare access emerged as a significant factor, with limited healthcare resources in rural areas contributing to higher infant mortality rates. This underscores the need for better healthcare access, especially in underserved areas, as a means to improve maternal and child health outcomes.

### Limitations and future recommendations

Among the shortcomings of the study are its cross-sectional design due to its reliance on self-reported data, which could skew results. The sample was also restricted to Khyber Pakhtunkhwa mothers, so the results might not be applicable to other areas. Future studies should use objective lifestyle and mental health measurements to lower bias, include a more varied sample from several areas of Pakistan, and examine longitudinal designs to prove causality. Additional research could look at how other social factors including education and employment affect maternal and child health results.

## Data Availability

The raw data supporting the conclusions of this article will be made available by the authors, without undue reservation.
